# Millimeter Wave Attenuation Due to Wind and Heavy Rain in a Tropical Region

**DOI:** 10.3390/s23052532

**Published:** 2023-02-24

**Authors:** Ukrit Mankong, Pakawat Chamsuk, Sitthichok Nakprasert, Sangdaun Potha, Zu-Kai Weng, Pham Tien Dat, Atsushi Kanno, Tetsuya Kawanishi

**Affiliations:** 1 Department of Electrical Engineering, Faculty of Engineering, Chiang Mai University, Chiang Mai 50200, Thailand; 2 Biomedical Engineering Institute, Chiang Mai University, Chiang Mai 50200, Thailand; 3 Center of Excellence in Quantum Technology, Faculty of Engineering, Chiang Mai University, Chiang Mai 50200, Thailand; 4 National Institute of Information and Communications Technology, Koganei 184-8795, Japan; 5 Nagoya Institute of Technology, Nagoya 466-8555, Japan; 6 Department of Electronic and Physical Systems, Faculty of Science and Engineering, Waseda University, Tokyo 169-8555, Japan; 7 Waseda Research Institute for Science and Engineering, Tokyo 169-8555, Japan

**Keywords:** fixed wireless link, millimeter wave, link budget, attenuation, tropical region

## Abstract

Millimeter wave fixed wireless systems in future backhaul and access network applications can be affected by weather conditions. The losses caused by rain attenuation and antenna misalignment due to wind-induced vibrations have greater impacts on the link budget reduction at E-band frequencies and higher. The current International Telecommunications Union Radiocommunication Sector (ITU-R) recommendation has been widely used to estimate rain attenuation, and the recent Asia Pacific Telecommunity (APT) report provides the model to estimate the wind-induced attenuation. This article provides the first experimental study of the combined rain and wind effects in a tropical location using both models at a frequency in the E band (74.625 GHz) and a short distance of 150 m. In addition to using wind speeds for attenuation estimation, the setup also provides direct antenna inclination angle measurements using the accelerometer data. This solves the limitation of relying on the wind speed since the wind-induced loss is dependent on the inclination direction. The results show that the current ITU-R model can be used to estimate the attenuation of a short fixed wireless link under heavy rain, and the addition of wind attenuation via the APT model can estimate the worst-case link budget during high wind speeds.

## 1. Introduction

Fixed wireless links using millimeter waves (mm-waves) and terahertz (THz) have been considered for use in both fifth-generation (5G) and sixth-generation (6G) wireless systems, for example, in the applications of point-to-point links in the backhaul and access networks [1,2,3,4,5]. The advantage of mm-waves and THz frequencies compared with microwaves is their wider bandwidths allowing for high-speed data transmissions. The frequencies in the 71–76 GHz and 81–86 GHz bands have been considered for commercial usage in the APT-AWG-REP-81 report [6]. Although, the path loss of mm-waves and the THz Fixed Wireless System (FWS) is larger due to their shorter wavelength and gaseous attenuations, the loss can be compensated by increasing the antenna gains, resulting in a narrow beam. Thus, not only the received signal strength of the FWS suffers from droplet attenuation, but the beam misalignment caused by a strong wind also causes fluctuation in the received signal strength in these high frequency bands. In a reverse manner, the variation of signal strengths in the FWS networks can be applied to sense weather conditions. The existing dense mm-wave links may be used to provide high resolution and real-time weather information that is unattainable using weather radars [7,8,9]. A comprehensive list of research works in rainfall measurements based on commercial microwave links was summarized in a recent review paper [10].

Wireless channel modeling may be achieved via machine learning techniques [11,12]. In a line-of-sight case, however, the attenuation is conventionally determined from physical phenomena including wave scattering, atmospheric absorption, and from empirical data such as ITU-R models. The process to estimate the attenuation involves, firstly, the calculation of specific attenuation in dB/km using the ITU-R P.838 model [13]. Then the attenuation for a specific distance is found by multiplying the specific attenuation by the effective path length, which is the product of the path length and the distance factor according to the ITU-R P.530 recommendation [14]. Recently, various works have been conducted to verify the standard models of rain attenuation given via ITU-R in several microwave, mm-wave and THz bands. Experimental studies found that no single model is suitable for all climate regions [15,16]. For example, the measurements in tropical Malaysia in 26 GHz and 32.6 GHz frequencies showed that the estimated attenuation using the ITU-R model underestimated the higher rain rate [17,18,19]. In the E band, there have been a few studies in tropical regions, such as long term measurements at 73 GHz and 83 GHz in Malaysia [20,21] and 71 GHz and 81 GHz in eastern China [9]; however, only rain attenuation was considered in the previous studies. In other works, the effect of rain on short-range fixed links have been investigated in the 25.84 GHz (K band) and 77.52 GHz (E band) bands in the United Kingdom, which has a temperate climate [22,23]. It was found that the updated ITU-R P.530-18 model provides the rain attenuation estimates that more closely match the experimental data when the path length is under 200 m than the previous version of the standard; however, the data were limited to rain rates under 30 mm/h. In the higher frequency bands, there have been experimental studies at 120 GHz [24] and 300 GHz [25,26] frequencies, but all in temperate climates. Only recently, there has been a study to evaluate the FWS link budget from both rain and wind effects both in the mm-wave region (theoretically and experimentally, in Japan) and the THz region (theoretically) [27]. Their results formed the basis on which the model in the APT-AWG-REP-81 report was developed [6]. The data for the wind-induced attenuation are still limited, especially when the wind effect combines with the rain effect simultaneously.

In this paper, we studied the concurrent rain and wind effects, which is the first study of this kind for E-band FWS links in a tropical location. To date, the only wind-induced attenuation model, caused by antenna vibration, relied on the experimental data in Japan [27], which has much different weather systems. Thus, the new data obtained in this paper present a significant contribution to assessing the applicability of combined rain and wind-induced attenuation models in new locations. During simultaneous wind and rain conditions in stormy weather, it was unclear whether the experimental data agree with either the current models of rain attenuation in the ITU-R P.530-18 recommendation [14] or the wind-induced attenuation in the APT-AWG-REP-81 model [6]. In addition, the wind effect as described in the APT report and the previous paper [27] used a wind speed sensor which showed statistical dependence between the wind speed and the received signal strength. However, several outliers were observed that were different from the expected values. This paper also shows that, by measuring the dynamic pole inclination angle using an improved setup to obtain accelerometer data, the effects of pole vibration on the link performance can be analyzed in more detail than that that was previously available. We collected data using the improved setup at Chiang Mai University, in Chiang Mai, Thailand during the rainy season of 2022. The data were analyzed against the aforementioned models. This paper is organized as follows. Section 2 presents the experimental setup with equipment specifications. Section 3 presents the link budget theory and calculation methods. Section 4 presents the results and analysis. Finally, Section 5 is the conclusions.

## 2. Experimental Setup

**Figure 1:**
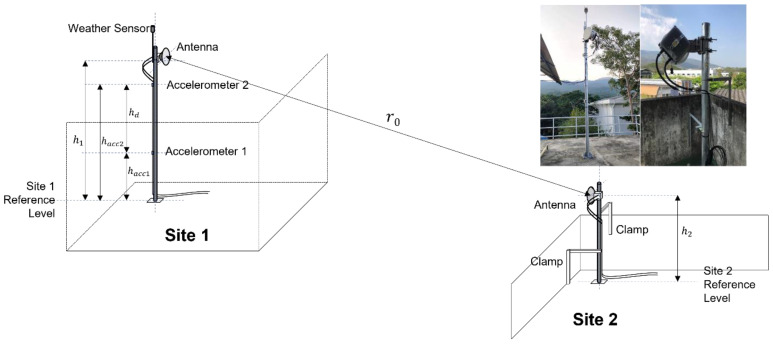
The experimental setup of the mm-wave FWS with antennas and poles. The accelerometers and weather sensor were installed at Site 1 where the pole vibration can be measured.

Figure 1 shows the experimental setup of the mm-wave FWS in Thailand. The poles in the setup were made using stainless steel (SS400) with a diameter of 101.6 mm (4 inches) and a thickness of 3.2 mm. The height ($h_{1}$) of an antenna of site 1 was 3.5 m; the total pole length was 4.0 m. Two accelerometers were attached to the pole at heights of 1.5 m and 3.0 m from the base. A weather logger for collecting wind, temperature and rain information was set on the top of the pole. The height ($h_{2}$) of the antenna of site 2 was 1.5 m; the total pole length was 2 m with lateral clamps. Thus, the pole deflection at site 2 should be negligible. The distance between the two sites was $r_{0}=150$ m.

**Figure 2:**
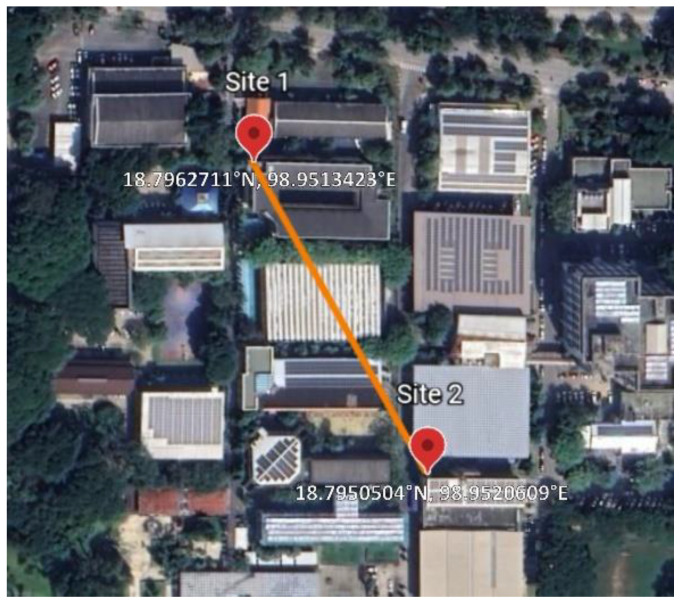
The map of experimental locations with their GPS coordinates.

The map of the experimental area is shown in Figure 2. The locations of the antennas (GPS coordinates 18.7962711° N, 98.9813423° E and 18.7959594° N, 98.9520609° E) were on the roofs of the two tallest buildings (4 stories). There was a line of sight (LOS) between the two antennas. We have analyzed in the supplementary materials, Figure S4 and Table S1, that the primary Fresnel zone was well clear of any obstruction, thus multipath interference may be ignored.

**Figure 3:**
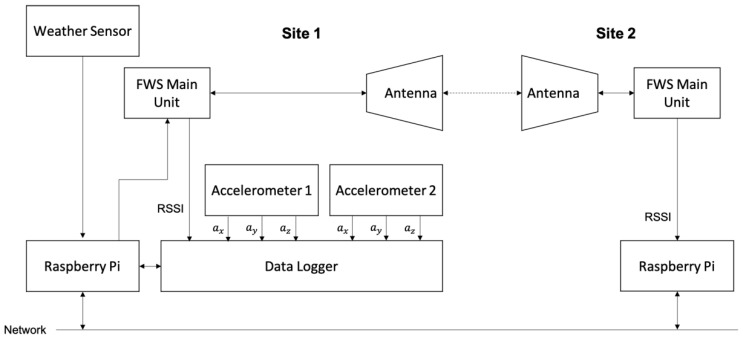
Schematic illustration of the antennas/sensors setup and block diagram of connections.

The equipment and their connections are also shown in Figure 3. Acceleration information obtained via the accelerometers, which has three-axis sensitivity, was captured via a data logger. The received signal strength indicator (RSSI) of the FWS main unit was also stored in the data logger in every 10 ms. The data logger was controlled via a single board computer such as Raspberry Pi. The weather data sensed by the weather logger were directly captured by the computer in every 10 s. In the other site, only the RSSI information was acquired via another single board computer, which controlled the FWS unit. All the data were transported via the Ethernet network to a data server.

The followings are the specifications of the equipment. The FWS unit is NEC iPASOLINK EX Advanced, with a minimum Tx power of −5 dBm and frequencies of 71–76 GHz and 81–86 GHz. The carrier frequency *f* = 74.625 GHz of the link from site 2 to site 1 was selected in the experiment. Automatic gain control (AGC) was turned off so that real-time attenuation due to weather effects could be measured. The antennas are COMMSCOPE-VHLP1-80 with a diameter of 0.3 m, beamwidth of 0.9°, 43.5 dBi gain, 14.0 dB return loss and a Voltage Standing Wave Ratio (VSWR) of 1.50. The total weight of each antenna and its mounting bracket is 7 kg. The pair of accelerometers (IMV-VP-8013M) measured 3-axis accelerations with a sensitivity of 44.9 mV/(m/s^2^). Their responses are in a range of 0.04–1000 Hz. The maximum measurable acceleration is ±58.8 m/s^2^. The RSSI level from the FWS unit and accelerometer data were sampled via the data logger (GRAPHTEC-GL980 midi LOGGER) at 10 ms intervals. Each data logger input has a 16-bit ADC with 0–30 V input range and 0–200 kHz frequency response.

The weather sensor (VAISALA-WXT536), located at site 1, can measure barometric pressure, air temperature, relative humidity, precipitation, and wind. The precipitation sensor has a collection area of 60 cm^2^ and can measure the rain rate in a range of 0–200 mm/h. Each value of the rain rate is a running 1-min average in 10-s intervals. The wind sensor can measure the wind speed in the range 0–60 m/s at a resolution of 0.1 m/s, with a response time of 0.25 s. Wind direction information can also be gathered with ±3° accuracy at 10 m/s. The weather sensor data were sampled at 10-s intervals using a Raspberry Pi.

## 3. Theory and Algorithms

### 3.1. FWS Link Budget

Based on the E-band FWS experiment setup in this work, the signal-to-noise ratio should be affected by antenna gains, free-space loss, gaseous loss, rain attenuation and attenuation via wind effect, given using

(1) $$\begin{array}{cc} \mathrm{SNR}(\mathrm{dB})=P_{tx} & +G_{t}+G_{r}\\ & -A_{free}-A_{gas}-A_{rain}-A_{wind}-N_{thermal}-N_{system}-M, \end{array}$$

where $P_{tx}$ is the transmit power (dBm), $G_{t}$ and $G_{r}$ are the gains (dBi) of the transmitter and receiver antennas, respectively, $A_{free}$ is the free space attenuation (dB), $A_{gas}$ is the gaseous attenuation (dB), $A_{rain}$ is the rain-induced attenuation (dB), $A_{wind}$ is the wind-induced attenuation (dB), $N_{thermal}$ is the thermal noise level (dB), $N_{system}$ is the system noise figure (dB) and M is the link margin (dB).

The first three parameters, the transmit power ($P_{tx}$), the transmitter antenna gain ($G_{t}$) and the receiver antenna gain ($G_{r}$), are constant, given via the user setting and the datasheet as shown in Table 1. The antenna gains of the 30 cm antennas at both sites are $G_{t}=G_{r}=43$ dB [28].

The next parameter, the free space attenuation, is determined by the frequency and path length:

(2) $$A_{free}=20\log_{10}\left(\frac{4\pi fr_{0}}{c}\right),$$

where f is the carrier frequency (Hz), $r_{0}$ is the path length (m) and c is the speed of light (m/s). At a frequency of *f* = 74.625 GHz and distance of *r*_0_ = 150 m, then $A_{free}=113.4$ dB.

The fourth parameter, gaseous attenuation, is calculated using ITU-R P.676 recommendation [29], as follows:

(3) $$A_{gas}=(\gamma_{o}+\gamma_{w})r_{0}=0.1820f\left(N_{\mathrm{Oxygen}}^{\prime \prime }(f)+N_{\mathrm{Water}\;\mathrm{vapour}}^{\prime \prime }(f)\right)\cdot r_{0},$$

where $\gamma_{o}$ and $\gamma_{w}$ are the specific attenuations (dB/km) due to dry air and water vapor, respectively, f is the frequency (GHz) and $r_{0}$ is the link distance (km). The imaginary parts of the frequency-dependent complex refractivities, $N_{\mathrm{Oxygen}}^{\prime \prime }(f)$ and $N_{\mathrm{water}\;\mathrm{vapour}}^{\prime \prime }(f)$, are calculated using

(4) $$N_{\mathrm{Oxygen}}^{\prime \prime }(f)=\sum_{i}S_{io}F_{io}+N_{D}^{\prime \prime }(f),$$

(5) $$N_{\mathrm{water}\;\mathrm{vapour}}^{\prime \prime }(f)=\sum_{i}S_{iw}F_{iw},$$

where $S_{io}$, and $S_{iw}$ are the strengths of the *i*th oxygen and water vapor lines, respectively, and $F_{io}$ and $F_{iw}$ are the oxygen and water vapor line shape factors, respectively. $N_{D}^{\prime \prime }$ is the dry continuum due to pressure-induced nitrogen absorption and the Debye spectrum. The oxygen and water vapor line strengths are given using

(6) $$S_{io}=a_{1}\times 10^{-7}\cdot p_{\mathrm{dry}\;\mathrm{air}}{\left(300/T\right)}^{3}\exp\left[a_{2}\left(1-300/T\right)\right],$$

(7) $$S_{iw}=b_{1}\times 10^{-1}\cdot p_{\mathrm{water}\;\mathrm{vapour}}{\left(300/T\right)}^{3.5}\exp\left[b_{2}\left(1-300/T\right)\right],$$

where $p_{\mathrm{dry}\;\mathrm{air}}$ is the dry air pressure (hPa), $p_{\mathrm{water}\;\mathrm{vapour}}$ is the water vapor partial pressure (hPa) and T is the temperature. The parameters $a_{1}$, $a_{2}$, $b_{1}$ and $b_{2}$ are given in [29] for all dry air and water vapor absorption lines between 50 GHz to 1780 GHz, which must be summed in Equations (4) and (5). The water vapor partial pressure can be estimated using the ITU-R P.835-6 recommendation [30] at a given altitude using

(8) $$p_{\mathrm{water}\;\mathrm{vapour}}(h)=\frac{\rho (h)T}{216.7}=\frac{\rho_{0}\exp(-h/h_{0})\;T}{216.7},$$

where $\rho_{0}=7.5$ g/m^3^ is the standard ground-level water vapor density, h is the location altitude (km) and $h_{0}=2$ km is the scale height. The dry air pressure can be found by subtracting the water vapor partial pressure from the total barometric air pressure ($p_{\mathrm{dry}\;\mathrm{air}}=p_{b}-p_{\mathrm{water}\;\mathrm{vapour}}$). The formulas for the line shape factors $F_{io}$ and $F_{iw}$, which also depend on the dry air and water vapor pressures, are given in detail in reference [29]. Thus, it can be seen from Equations (3)–(8) that by measuring the barometric air pressure ($p_{b}$) and the temperature (T) at the link location, the gaseous attenuation ($A_{gas}$) can be calculated.

**Figure 4:**
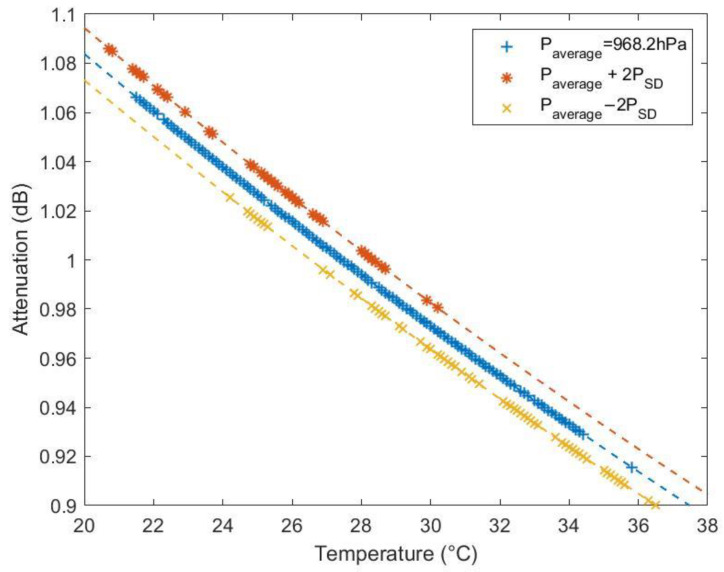
The range of gaseous attenuation calculated from the measured data.

The barometric air pressure and temperature data have been collected over an 8-month period in 2022, between March and October, at the experiment location. The average air pressure ($p_{average}$) is 968.2 hPa with a standard deviation of 2.5 hPa, and the temperature varies between 20.4 and 36.3 °C. During these months, the weather is governed by low-pressure systems and high temperatures. We calculated the gaseous attenuation at a link distance of 150 m as the function of temperatures at 3 air pressure values: $p_{average}$, $p_{average}+2SD$ and $p_{average}-2SD$, as shown in Figure 4. Thus, the value of $A_{gas}$ should fall in the range of 0.899–1.087 dB.

The final three parameters in Equation (1) are thermal noise, the total system noise figure and the margin, respectively. These parameters can be estimated for a given link as follows. The thermal noise is calculated as

(9) $$N_{thermal}=10\log_{10}\left(\frac{kBT}{1\;\mathrm{mW}}\right),$$

where k is the Boltzmann’s constant, B is the signal bandwidth (Hz) and T is the absolute temperature (K). $N_{system}$, the total noise figure of the system, is a constant value for a particular link. Finally, the parameter M is an additional margin of the link budget.

The values of losses in the link budget equation, excluding rain and wind-induced attenuation, are summarized in Table 1. These values are almost constant in the FWS link; thus, they have little effects on the dynamic performance. On the other hand, the losses due to rain ($A_{rain}$) and wind ($A_{wind}$) greatly depend on weather conditions, which will be discussed in the following sections.

### 3.2. Rain Attenuation

The rain attenuation can be calculated from the rain rate using the empirical model in the ITU-R P.838-3 recommendation [13,14]. The specific attenuation ($\gamma_{R}$) and the total attenuation ($A_{rain}$) in dB is given using

(10) $$A_{rain}=\gamma_{R}r_{eff}=k_{p}R^{\alpha_{p}}\cdot rr_{0},$$

where R is the rain rate (mm/h), $k_{p}$ is the coefficient of either vertical polarization ($k_{V}$) or horizontal polarization ($k_{H}$), $\alpha_{p}$ is the coefficient of vertical polarization ($\alpha_{V}$) or horizontal polarization ($\alpha_{H}$), $r_{eff}=rr_{0}$ is the effective path distance between two antennas and r is the distance factor.

The distance factor r is determined using the ITU-R P.530-18 recommendation [14], given as

(11) $$r=\frac{1}{0.477r_{0}{}^{0.633}R_{0.01}^{0.073\alpha }f^{0.123}-10.579\left[1-\exp(-0.024r_{0})\right]},$$

where $R_{0.01}$ is the 1-min rain rate exceeded for 0.01% of the time. The rain rate $R_{0.01}$ is determined from the cumulative distribution function (CDF) of rain rates, collected over a long period. In the case that rain statistics cannot be measured, an estimate can be calculated from the ITU-R P.837-7 recommendation [31]. The formula of the distance parameter was derived empirically. In the previous ITU-R P.530-17 release, the factor was limited to the maximum value of 2.5. However, experimental data from previous studies have shown that for short distant links (under 1 km) such a limit resulted in the underestimation of rain attenuation [22,32]. For the current ITU-R P.530-18 model used in this study, the maximum limit of r has been removed. Using the values for 74.625 GHz and vertical polarization, $k_{p}=k_{v}=$ 1.0946 and $\alpha_{p}=\alpha_{v}=$ 0.7118. The $R_{0.01}$ rain rate measured in 2022 at the experimental location is $R_{0.01}=$ 83.2 mm/h, and this yields r= 3.7075.

The specific attenuation in Equation (10) is determined using the rain rate. However, previous studies have shown that smaller rain drop sizes, associated with lower rain rates, cause higher attenuations in the E band [33]. Thus, without measuring the actual rain drop size distribution via an instrument such as a raindrop spectrometer, the ITU-R P.838 model may underestimate the specific attenuation. In a tropical location, the power budget needs to be designed for larger rain rates; then, the model should be sufficient.

### 3.3. Inclination Attenuation Due to Wind

**Figure 5:**
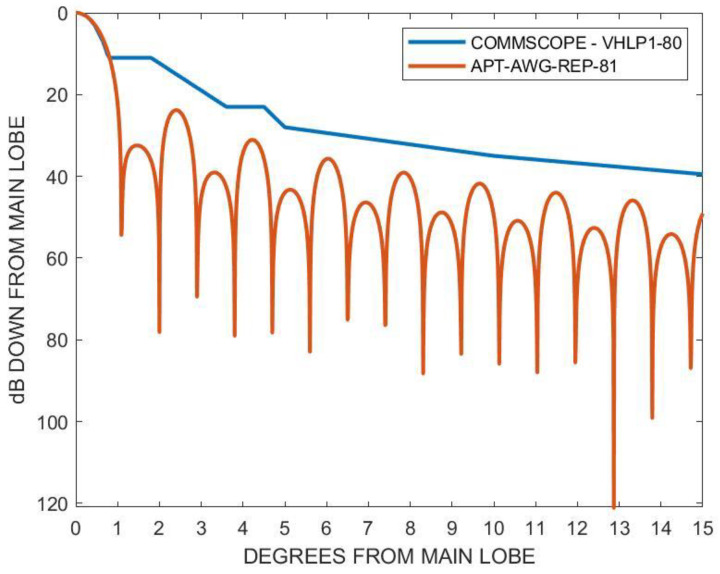
Radiation pattern envelope as a function of the misalignment angle using Equation (12) compared to the manufacturer’s data.

Windy conditions cause dynamic load on the pole and antenna structure, leading to dynamic inclination and antenna misalignment. It has been shown in the APT-AWG-REP-81 report [6] that the gain degradation depends on the beam misalignment angle, given as

(12) $$A_{wind}=g(\theta )=20\log_{10}\left(\frac{u^{\prime }}{2J_{1}(u^{\prime })}\right),\;\left(\mathrm{for}\;\frac{D}{\lambda }\leq 100\right)$$

where g(θ) is the gain degradation in dB as a function of the misalignment angle (θ), $J_{1}$ is the Bessel function of the first kind, D is the diameter of the antenna and λ is the wavelength. The parameter $u^{\prime }$ is given using

(13) $$u^{\prime }=\frac{60\pi }{\theta_{BW}}\sin\theta ,$$

where $\theta_{BW}=k\lambda /D$ is the half-power beamwidth and k is the factor depending on the shape of the reflector and the feed illumination pattern. Alternatively, angular gain degradation can be estimated from the antenna manufacturer. For example, the COMMSCOPE–VHLP1-80 antenna has online data that can be referred to [28]. Figure 5 shows the radiation pattern envelope of the antenna, calculated using Equations (12) and (13), compared to the manufacturer’s data. It should be noted that the gain is based on the angular deflection of the antenna, where the pole is deflected in the pitch direction with respect to the antenna front. The pole inclination can also occur in the side directions where the beam misalignment angle is much smaller. In such cases, the gain degradation is smaller than the calculated value (see the Supplementary Materials, Figures S1–S3).

Precise measurement of the inclination angle allows for an accurate estimation of the link performance in the parameter $A_{wind}$, so that effective mitigation can be applied. The method presented in APT-AWG-REP-81 [6] and Z.K. Weng et.al. [27] used the wind speed to calculate the wind load-induced inclination based on the structural properties of the pole and antenna. Their findings showed that the results agree with the model, which predicts that the angle is proportional to the square of wind speed, and that the attenuation is dependent on the wind speed. Another method to measure the inclination is the direct measurement of the inclination angle from the accelerometer data in our setup in Figure 1. The time-varying 3D angle can be obtained, which can provide more insights into the dynamic of wind-induced attenuation. The two methods to estimate the pole inclination are summarized in the following subsections.

#### 3.3.1. Dynamic Pole Angle Estimation via Wind-Induced Mechanical Vibration

**Figure 6:**
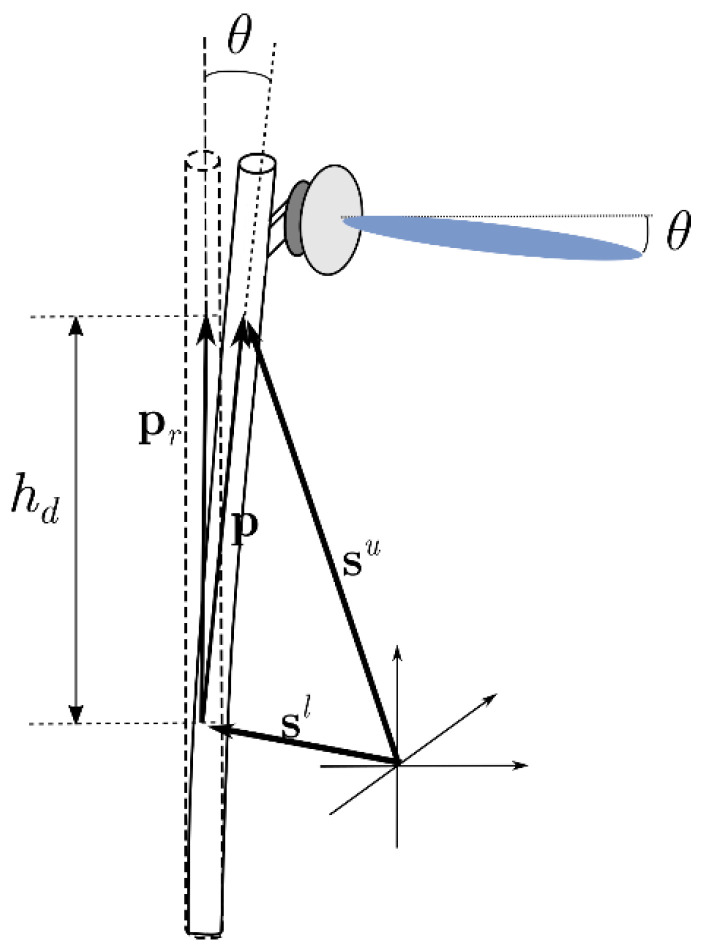
3D inclination angle measurement diagram.

Pole inclination, θ, in the direction of the antenna front is equal to the angular misalignment of the beam, as shown in Figure 6. The estimation of the pole inclination is based on the theory presented in the APT-AWG-REP-81 report [6] and a previous study [27]:

(14) $$\theta =\theta_{0}+\theta_{s}+\theta_{d},$$

where $\theta_{0}$ is the initial axis misalignment, $\theta_{s}$ is the static inclination angle and $\theta_{d}$ is the dynamic inclination angle. The static inclination angle is caused by the assumed constant wind load and is given using

(15) $$\theta_{s}=(C_{1}A_{1}+3C_{2}A_{2})\frac{\rho l^{2}}{12EI}v^{2}=C_{s}v^{2}.$$

The dynamic inclination is caused by the natural vibration frequency of the pole and is given using

(16) $$\theta_{d}=C_{d}v^{2}.$$

The wind load coefficients for static and dynamic inclination, $C_{s}$ and $C_{d}$, depend on the type and dimensions of the pole, respectively. v is the effective wind speed (m/s), $C_{1}$ and $A_{1}$ are the drag coefficient and the wind receiving area of the pole, respectively, $C_{2}$ and $A_{2}$ are the drag coefficient and the wind receiving area of the antenna, respectively, ρ is the air density (kg/m^3^), l is the length of the pole (m), E is the Young’s modulus and I is the second moment of area (m^4^).

For this setup in Figure 1, the parameters and their values used for angle calculation are given in Table 2. The pole length and diameter are used to calculate the wind-receiving area of the pole ($A_{1}$). The antenna diameter is used to calculate the wind-receiving area of the antenna ($A_{2}$). The drag coefficients $C_{1}$ and $C_{2}$ are determined by $A_{1}$ and $A_{2}$, respectively, using [34] and taking into account the cylindrical and flat shapes of the pole and antenna. The value of air density, ρ= 1.124 kg/m^3^, is calculated from the average temperature and pressure using the ideal gas law. The Young’s modulus, E= 200 × 10^9^ Pa, is typical of stainless steel SS400. The second moment of area can be calculated to be 1.8 × 10^−4^ m^4^ for the annulus cross-section of the pole [35].

#### 3.3.2. Direct Dynamic Pole Angle Measurements via Accelerometer

The dynamic pole inclination angle can be directly measured using the setup in Figure 1. The measured accelerometer data consist of 3-axis accelerations at the lower and upper positions on the pole. The sampling period for the acceleration data was 10 ms. The accelerations were high-pass filtered at the frequency below the natural vibration frequency of the pole to remove the gravity and the initial inclination effects. Let the filtered accelerations of the lower and upper sensors be $a_{x}^{l},\;a_{y}^{l},\;a_{z}^{l}$ and $a_{x}^{u},\;a_{y}^{u},\;a_{z}^{u}$, respectively. The double integration of accelerations yields the time-varying displacement of both pole positions,

(17) $$s^{q}=\iint \left(a_{x}^{q}\hat{x}+a_{y}^{q}\hat{y}+a_{z}^{q}\hat{z}\right)\;d\tau d\tau^{\prime }$$

where q=l, u denotes the upper and lower sensor positions. The pole vector is $p=s^{u}-s^{l}$, as shown in Figure 6. The inclination angle magnitude is calculated from the dot product using

(18) $$\theta =\cos^{-1}\left[(p\cdot p_{r})/h_{d}^{2}\right]$$

where $p_{r}$ is the optimal pole position and $h_{d}$ is the height difference of the two accelerometers. Alternatively, the directional 3D inclination angle can be calculated using the cross product

(19) $$\theta =\sin^{-1}\left[\left\|p\times p_{r}\right\|/h_{d}^{2}\right]\hat{n}$$

where $\hat{n}$ is the unit vector in the direction of $p\times p_{r}$. Comparing to Section 3.3.1, the angle obtained in Equations (18) or (19) should be equal to the sum of initial axis misalignment, and static and dynamic angles due to wind, as in Equation (14).

## 4. Results and Analysis

### 4.1. The Distribution of Rain Rate and Wind Speed

**Figure 7:**
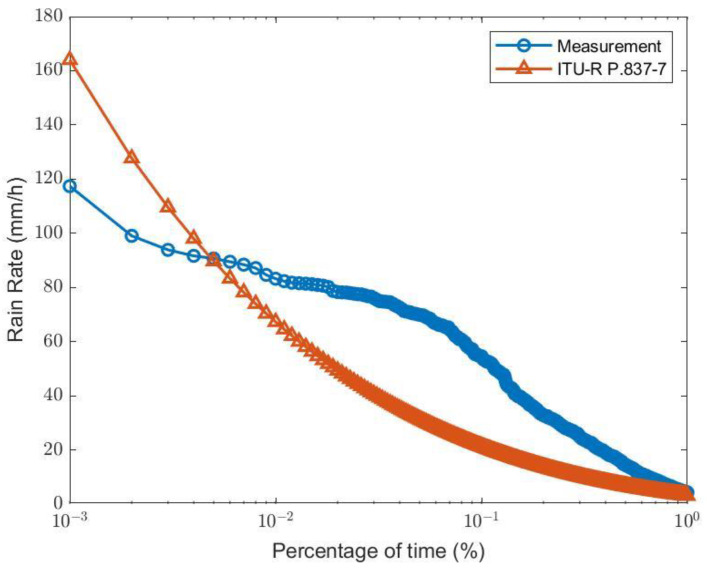
Cumulative distribution of the measured rain rates compared to the ITU-R P.837-7 model.

**Figure 8:**
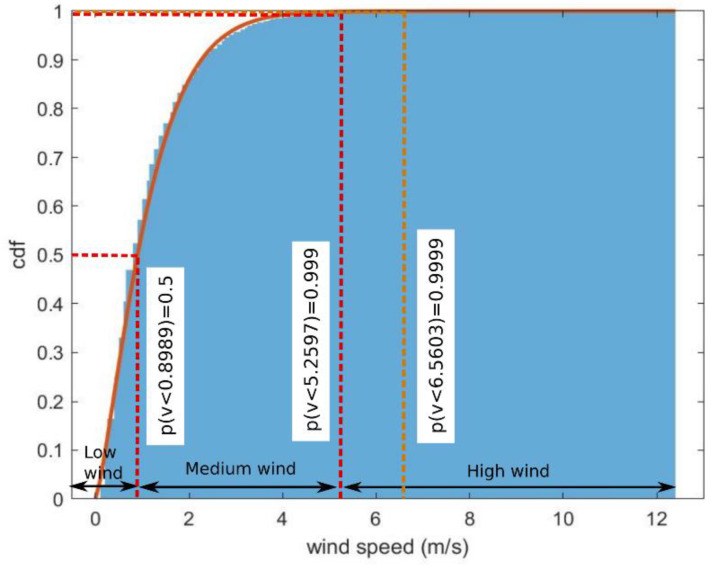
Wind speed cumulative distribution function (cdf) and Weibull distribution fit.

The results in Figure 7 and Figure 8 show rain and wind distributions over the 8-month period during the rainy season in 2022, at Chiang Mai University, Thailand. The data may be used for annual statistical prediction since the climate outside the period is dry. At the measurement location, which is a tropical location, the weather characteristics include heavy rain. This is seen in the cumulative distribution of the measured rain rates, as in Figure 7. The measured rain rate, which exceeded 0.01% of the time $R_{0.01}=$ 83.20 mm/h is used for analysis, and which is higher than the prediction given using the ITU-R P.837-7 recommendation [16] of $R_{0.01}=$ 67.27 mm/h.

The cumulative distribution function of the wind speed during the same period was plotted in Figure 8. As described in [27], the wind speed distribution can be modelled using a Weibull distribution:

(20) $$F(v)=1-\exp\left[-{\left(v/\eta \right)}^{\beta }\right],$$

where v is the wind speed (m/s), η is the scale factor and β is the shape factor. The value of β and η for the measured data are 1.1911 and 1.3016, respectively. The values of the wind speeds at 50%, 99.9% and 99.99% of the time were 0.8989 m/s, 5.2597 m/s and 6.5603 m/s, and are used as the low-wind threshold, the high-wind threshold and the value used in the link budget calculation, respectively.

### 4.2. Rain Attenuation and Combined Rain-Wind Attenuation

**Figure 9:**
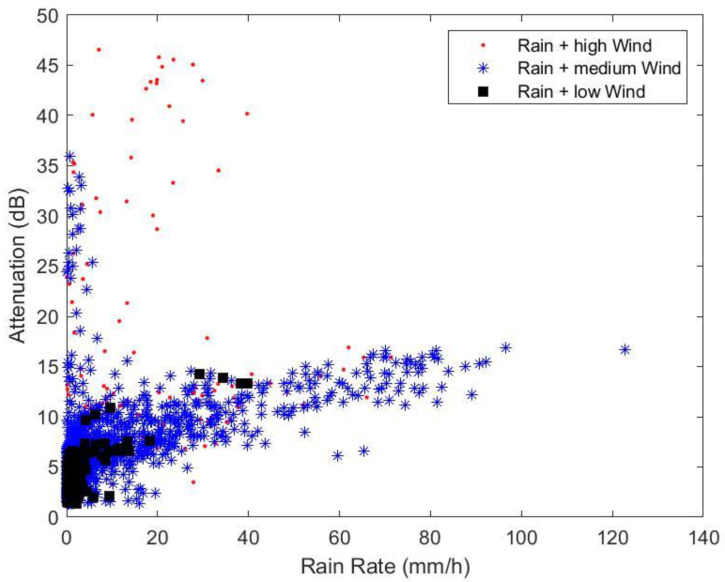
Maximum attenuation in 1-min intervals as a function of the rain rate of three groups of data: high wind speed, medium wind speed, and low wind speed.

One of the major sources of the attenuation in the FWS link budget calculation is the attenuation due to rain, as discussed in Section 3.1. The maximum attenuations within 1-min intervals have been plotted against the average rain rate in the same intervals, as in Figure 9. Only the intervals with non-zero detected rain falls have been included. The data are divided into three groups: low wind, medium wind and high wind, according to the maximum detected wind speed in each interval. Low wind, medium wind and high wind are defined as the wind speeds in the ranges of 0–0.8989 m/s, 0.8989–5.2597 m/s and >5.22597 m/s, respectively. The threshold values depend on the statistical distribution of the wind speed, which is assumed to follow the Weibull distribution; they are the wind speeds at 50% and 99.9% of the time. The majority of data in all groups show similar rain attenuation dependency with the rain rate. However, in the medium and high wind groups, we can observe occasional large attenuations up to 36 dB and 47 dB, respectively. The measurement data indicate that the performance of FWS at mm-waves and higher frequencies should consider the effect of wind-induced attenuation in addition to the rain attenuation. In addition, we have demonstrated that the wind speed criteria using the Weibull distribution suggested in [6] can be used to represent the data in calculating the effect of wind-induced attenuation.

**Figure 10:**
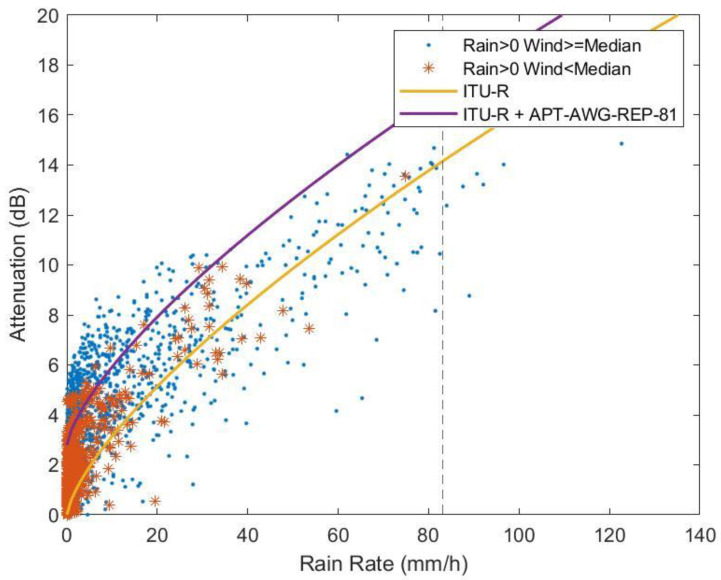
Average attenuation in 1-min intervals as a function of rain rate of two groups of data compared with ITU-R model and the combination of ITU-R and APT-AWG-REP-81 models.

In addition, we have compared the measured data with the ITU-R model for rain attenuation by using the following models: ITU-R P.838 [13], ITU-R P.530-18 [14] and APT-AWG-REP-81 [6]. In Figure 10, the data have been divided into two groups: rain with the top 50% of wind speeds, and rain with the bottom 50% of wind speeds. In contrast to Figure 9, the average attenuations within 1-min intervals have been used to comply with the model definition. The vertical dash line indicates the $R_{0.01}=$ 83.2 mm/h rain rate. The results show that the current version of ITU-R models provides a good estimate for the average attenuations when rain rates are close to the $R_{0.01}$ value. At low rain rates, on the other hand, there was significant wind-induced attenuation where the two groups differ. The combined rain model (ITU-R) and wind model (APT-AWG-REP-81), the purple line, provides a better estimation at low rain rates. Using a 99.99% wind speed to calculate the inclination angle as summarized in Section 3.3.1, the inclusion of APT wind attenuation model adds extra 2.782 dB of attenuation.

### 4.3. Wind-Induced Attenuation Using Direct Inclination Angle Measurement

**Figure 11:**
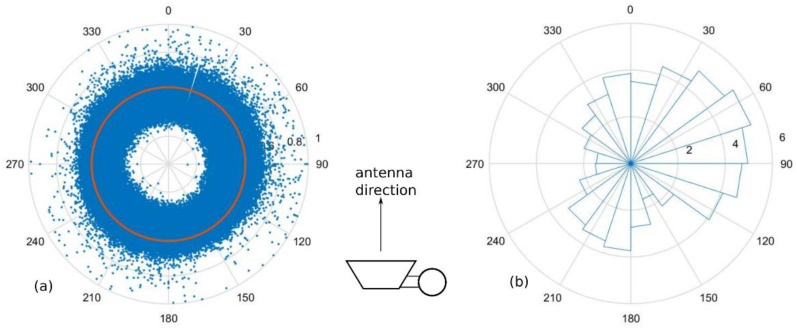
(**a**) Angle magnitude plot versus wind direction, with the threshold shown; (**b**) histogram of angle magnitudes that exceed threshold according to the wind direction (the number of samples ×1000). The antenna is facing 0°.

**Figure 12:**
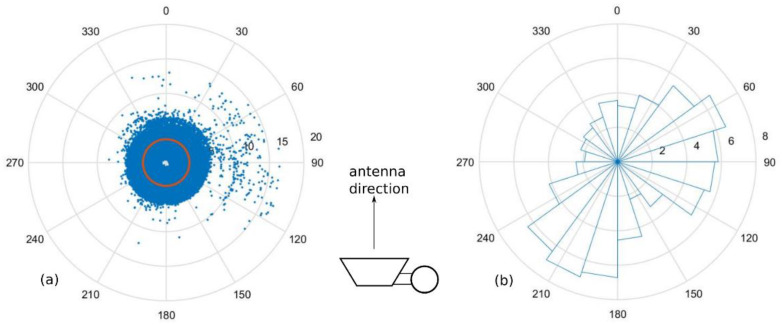
(**a**) Attenuation values plot versus wind direction, with threshold shown; (**b**) histogram of attenuation values that exceed threshold according to the wind direction (the number of samples ×1000). The antenna is facing 0°.

Firstly, the effect of wind direction is demonstrated. The plots of the inclination angle (*θ*) versus wind direction are shown in Figure 11, where Figure 11a is the angle magnitude and Figure 11b is the histogram of the angle magnitude exceeding the threshold of 0.5496° (Avg + SD). The plots of attenuation versus wind direction are shown in Figure 12, where Figure 12a contains all values of attenuation, and Figure 12b is the histogram of the attenuation exceeding the threshold of 3.3739 dB (Avg + SD). The top-view images of the pole and antenna orientation are also depicted, where the antenna is facing at 0°. It was observed that the inclination angle or the attenuation was larger in specific wind directions. Hence, only the wind speed data are not sufficient to estimate the attenuation, in contrast to the previous study [27], where the wind speed can solely be used to estimate the attenuation using Equations (12)–(16). This may be due to the off-centered weight of the combine pole and antenna unit as well as the effects of the weather system and the surroundings of the different field trial location.

**Figure 13:**
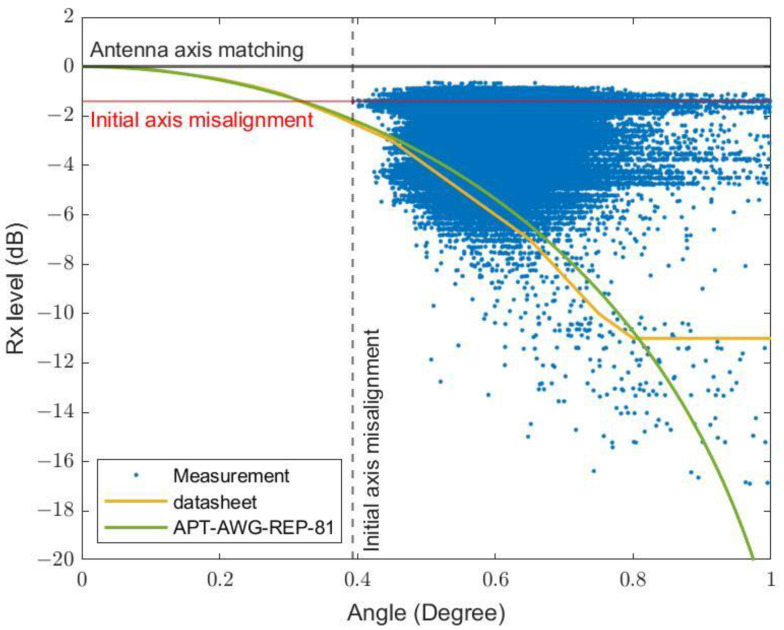
Minimum received signal versus inclination angle magnitude via direct measurement due to wind.

It is recommended to directly measure the inclination angle, for example using accelerometers, as described in Section 3.3.2, in order to assess wind-induced reduction across various geographical locations. In this experiment, we analyze the correlation between the maximum inclination angle and the minimum received power within 10-s intervals, for consistency with the previous report [6,27]. In the first data group in Figure 13, only the data with no rain (maximum rain rate = zero) in each interval have been included to eliminate the effect of rain attenuation. We see that both Equation (12) (APT-AWG-REP-81 model) and the datasheet can be used to estimate the lower limit of the wind-induced attenuation using a direct inclination angle measurement. From the measured data, initial axis misalignment of approximately 0.394° causes approximately 1.404 dB of attenuation, slightly lower than the expected attenuation of 2.16 dB from the model. Most data, however, do not show dependency with the pole inclination angle. This may be due to the inclination directions, which were not included in the model in Section 3. Side inclination of the antenna pole in this setup produced a much lower attenuation, by two orders of magnitude (see the Supplementary Materials, Figures S1–S3).

**Figure 14:**
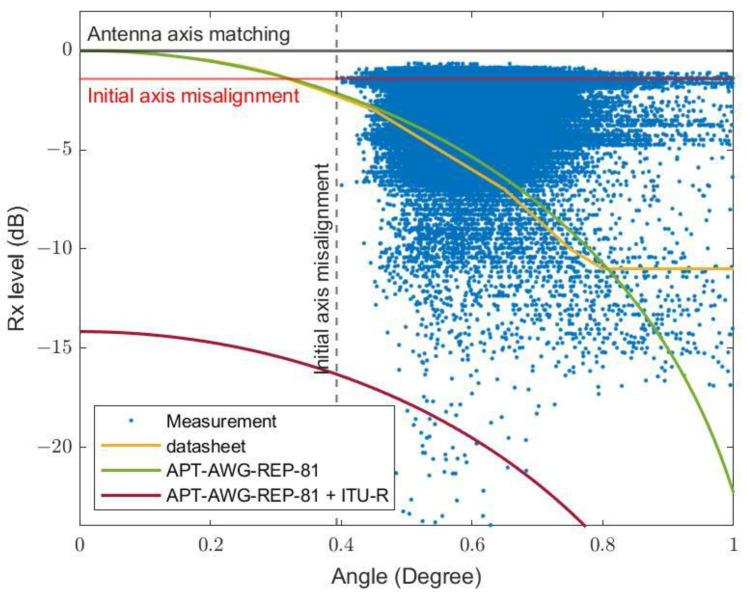
Minimum received signal versus inclination angle magnitude via direct measurement due to wind and rain.

In Figure 14, the data include those with positive maximum rain rates. Compared to Figure 13, there have been many data points with much lower than expected received powers, predicted using the APT-AWG-REP-81 model, especially at small inclination angles due to the additional rain attenuation. We have shown that by starting with the model for wind-induced attenuation under zero-rain conditions (the green curve), the effect of rain attenuation calculated using the ITU-R model using a $R_{0.01}$ rain rate (obtained in Figure 7) can be added. The combined model (the dark-red curve) can be used to estimate the lower limit of total attenuation.

### 4.4. Dependence on the Polarization and the Link Distance

**Figure 15:**
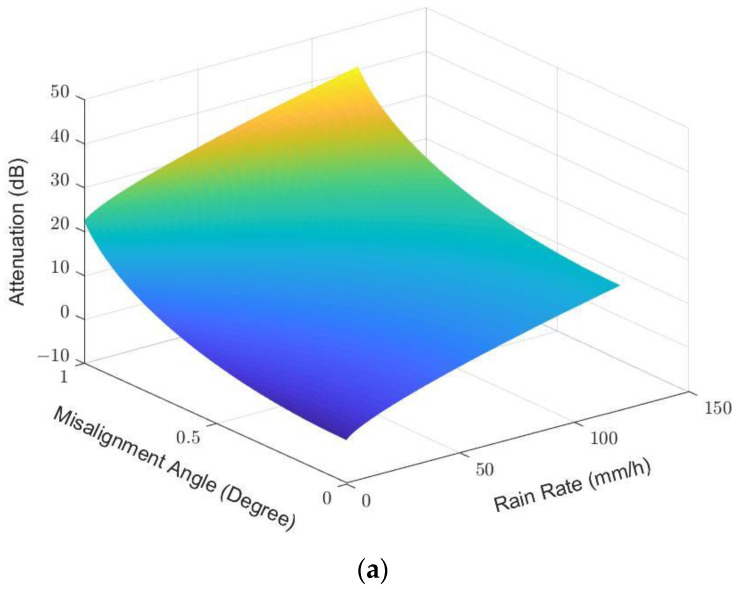

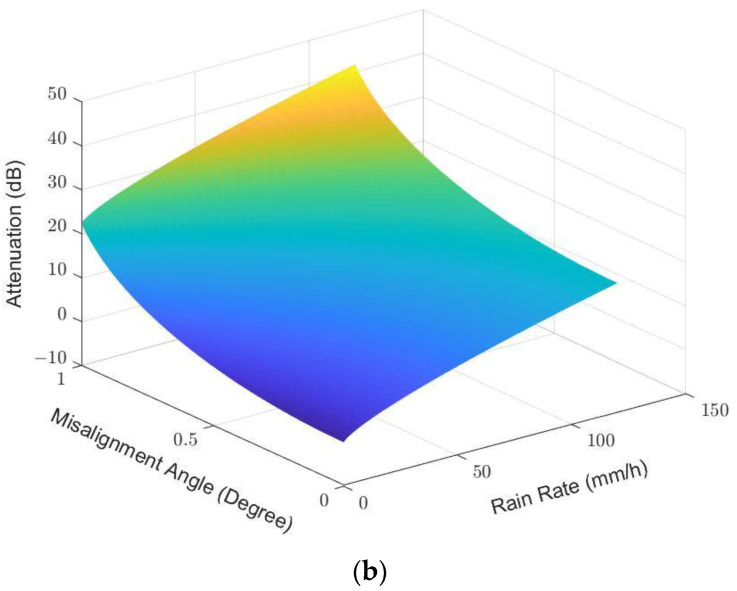
3D plot of the attenuation as the function of rain rate and misalignment angle for (**a**) the vertical polarization and (**b**) the horizontal polarization.

Having established from the results in Section 4.2 and Section 4.3 that the wind-induced attenuation can be added on top of the rain attenuation (and vice versa), we may apply the models to show dependency of the polarization and the link distance. Figure 15a,b show the attenuations at different rain rates and misalignment angles (caused by the wind effect) for the vertical and horizontal polarizations, respectively. An angle misalignment of 1 degree, due to wind effect coupling with the initial misalignment, could add as much as 20 dB of attenuation. The horizontal polarization attenuations were inferred from the available measurement data by changing the parameters $k_{H}=1.0996$ and $\alpha_{H}=0.7230$ in Equation (10), provided using the ITU-R P.838 standard. At the R0.01 rain rate and the distance $r_{0}=150$ m, the attenuation of the horizontal polarization attenuation is estimated to be 0.7244 dB higher than that of the vertical polarization. The difference is larger at heavier rain rates due to the oblate shape of the larger rain drop sizes.

**Figure 16:**
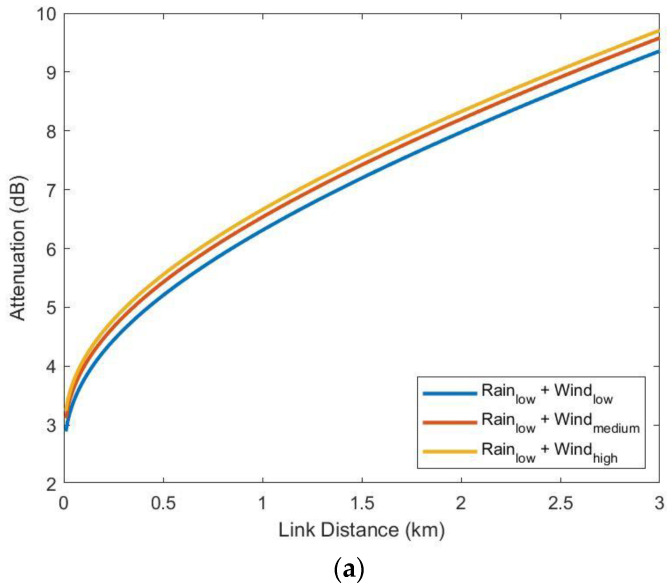

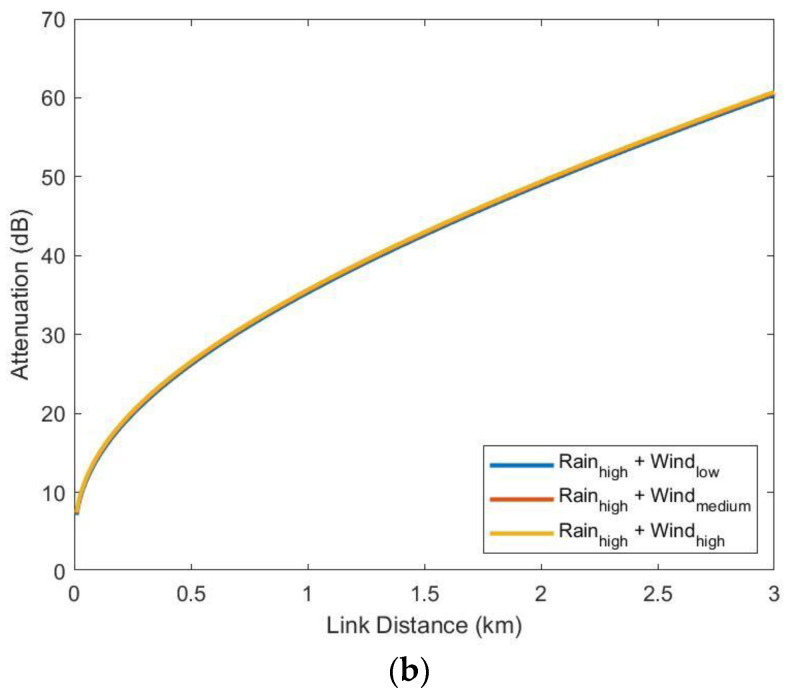
Expected attenuations as functions of the link distance under (**a**) low rain conditions and (**b**) high rain conditions.

The attenuations at longer path distances can also be estimated under various weather conditions, as shown in Figure 16a,b. In these two figures, the initial misalignment effect has been included, but the gaseous and free-space path losses are excluded. The attenuations caused by different wind speeds are significant at a low rain rate (the rain rate exceeded for 1% of the time, R = R_1_ = 4.3 mm/h), as seen in Figure 16a. On the other hand, at a high rain rate (R = R_0.01_ = 83.20 mm/h) in Figure 16b, the rain attenuations dominate the total attenuations. The values of low, medium and high wind speeds are the values at 0.5, 0.999 and 0.9999 of cumulative probability in Figure 8, respectively.

### 4.5. Antenna Misalignment and Realignment Due to Gust Events

Sudden changes in RSSI, such as a rising or dropping of up to 1–2 dB, have been observed occasionally after gusty periods. We have attributed this effect to misalignment and realignment caused by wind gusts. The definition of such misalignment is the decrease or increase in RSSI beyond the threshold ($\Delta P_{th}$) within a 5-min period. We calculated the probability of misalignment and realignment as functions of the maximum wind speed within the period. The algorithm searches the data to find the mis- and re-alignment events in each 5-min period where $\Delta RSSI\geq \Delta P_{th}$, while rain rate remains below $R_{\mathrm{th}}$ within the same period. $R_{\mathrm{th}}$ is the rain rate in mm/h, averaged over 1 min, such that the attenuation caused solely by rain is equal to $\Delta P_{th}$ according to ITU-R models. The exclusion of events where the rain rate exceeded the threshold is to ensure that the RSSI shifts were caused by wind gusts.

**Figure 17:**
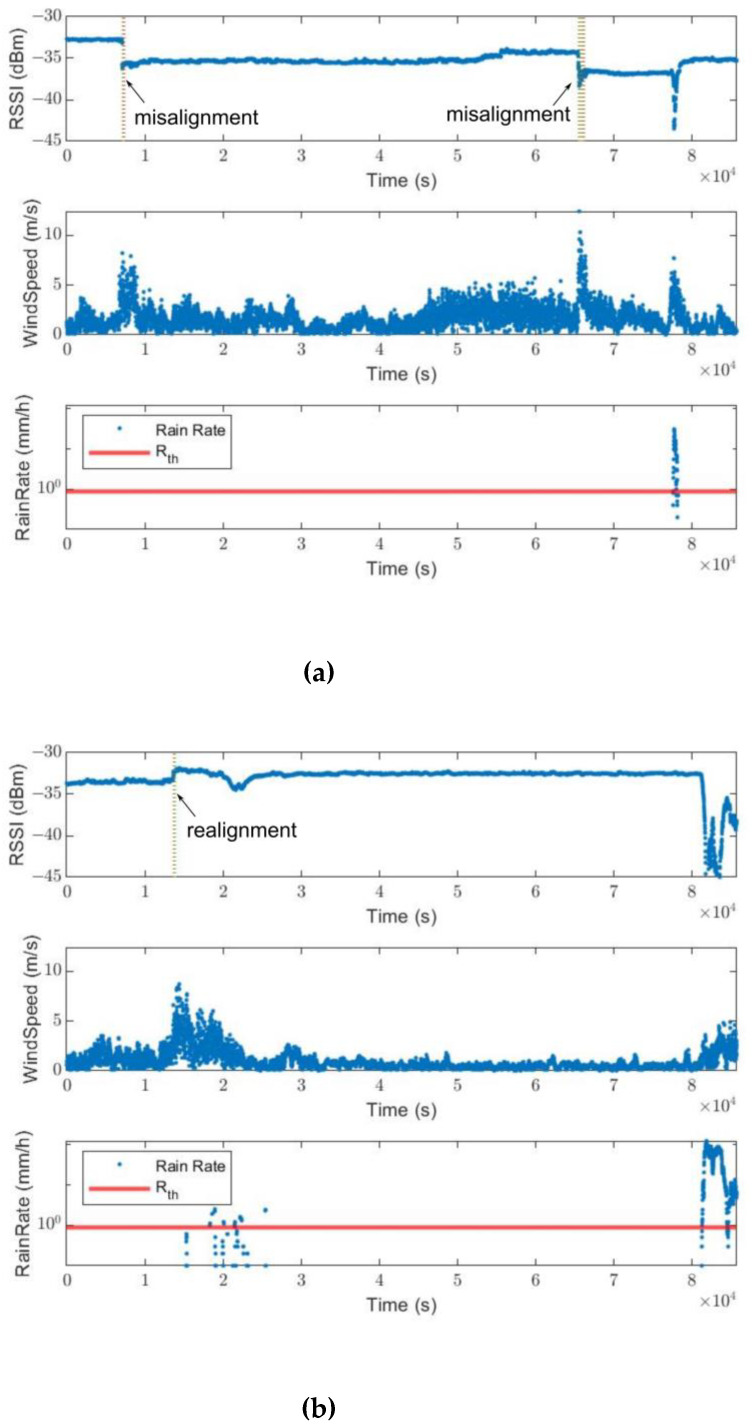
Examples of misalignment and realignment events caused by gust of wind causing either an increase or decrease in RSSI. (**a**) Examples of misalignment events, only RSSI drops while the rain rate is below *R*_th_ are counted. (**b**) Examples of realignment events, only RSSI increases while the rain rate is below *R*_th_ are counted.

**Figure 18:**
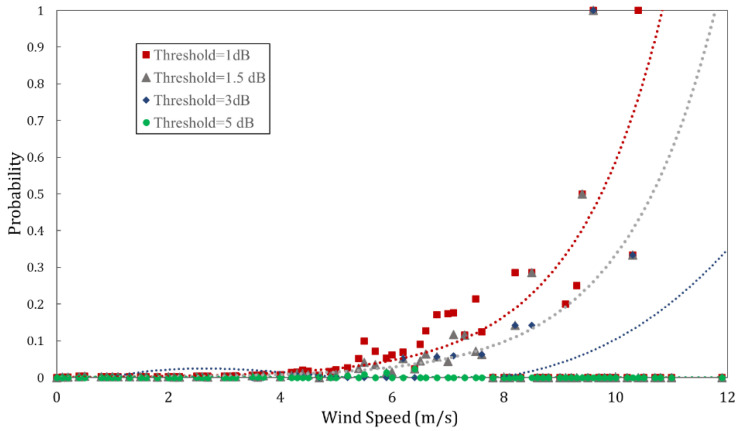
Probability of misalignment and realignment events due to wind gusts over the measurement period, at different RSSI shift thresholds.

As seen in Figure 17a,b, some instances of wind gusts worsen the antenna alignment (misalignment events), while the other instances improve the antenna alignment (realignment events). For a fixed value of RSSI shift threshold $\Delta P_{th}$ (1 dB, 1.5 dB, 3 dB or 5 dB), the probability of misalignment events increases with the maximum wind speed, as shown in Figure 18. However, no RSSI shifts of more than 5 dB threshold were observed. Hence, mechanical realignment may be required following strong wind.

## 5. Conclusions

This article presents the first field trial of the E-band FWS link in a tropical location which considers the combined rain attenuation and wind-induced attenuation. The data were analyzed using the current ITU-R P.530-18 rain attenuation model and APT-AWG-REP-81 wind-induced attenuation model. The data showed wind-induced attenuation is significant in this frequency region. By combining both models, the worst-case link budget could be estimated. Although the pole inclination angle and thus the wind-induced attenuation can be estimated via wind speed using the APT report, the direct inclination angle measurements enabling detailed weather effects which account for different inclination directions can be applied to new locations with different weather conditions. Further work can be performed to study the practical effects of wind and rain in FWS link performance, and to develop a compensation technique to ensure stable performance.

## Figures and Tables

**Table 1:** Values of parameters in the link budget equation.

Parameters	Values	Calculation Method/Reference
$\mathrm{Transmit}\;\mathrm{power}\;(P_{tx})$	−5 dBm	User setting
$\mathrm{Antenna}\;\mathrm{gains}\;(G_{t},G_{r})$	43 dB	Datasheet
Free-space loss ($A_{free}$)	113.4 dB	at *f* = 74.625 GHz and *r*_0_ = 150 m
$\mathrm{Gaseous}\;\mathrm{loss}\;(A_{gas})$	0.899–1.087 dB	ITU-R P.676 [29]

**Table 2:** Values of parameters for the calculation of inclination angle due to wind speed.

Symbol	Quantity	Value	Unit
$C_{1}$	Drag coefficient of the pole	0.98	-
$A_{1}$	Wind receiving area of the pole	0.36	m^2^
$C_{2}$	Drag coefficient of the antenna	1.42	-
$A_{2}$	Wind receiving area of the antenna	0.118	m^2^
ρ	Air density	1.124	kg/m^3^
l	Pole length	4	m
d	Pole diameter	101.6 (4)	mm (inch)
D	Antenna diameter	0.3 (1)	m (ft)
E	Young’s modulus	200 × 10^9^	Pa
I	Second moment of area	1.8 × 10^−6^	m^4^

## Data Availability

The data presented in this study are available on request from the corresponding author.

## Supplementary Materials

The following supporting information can be downloaded at: https://www.mdpi.com/article/10.3390/s23052532/s1. Figure S1: beam misalignment angle due to pitch inclination; Figure S2: beam misalignment angle due to side inclination; Figure S3: comparison of gain degradation by pitch and side inclinations; Figure S4: diagram of the actual link and existing buildings between the path; Table S1: primary Fresnel zone clearance data.
