# Supplementary material for: Millimeter Wave Attenuation Due to Wind and Heavy Rain in a Tropical Region
Source: Sensors (Basel). 2023 Feb 24;23(5):2532. doi: 10.3390/s23052532 (PMC10007627; doi:10.3390/s23052532)
Supplement: Supplementary file 1 [file sensors-23-02532-s001.zip › sensors-2131039-supplementary.pdf]

Supplementary materials

1. Analysis of attenuation caused by pole inclination in the pitch direction and the side direction (with respect to the LOS path direction).

The two inclination angles, pitch and side inclinations have different effects on the angular alignment of the beam.

Firstly, for the pitch inclination, it can be shown by geometry that the beam misalignment angle (φ_1) is equal to the pole inclination angle (θ) (see Figure S1)

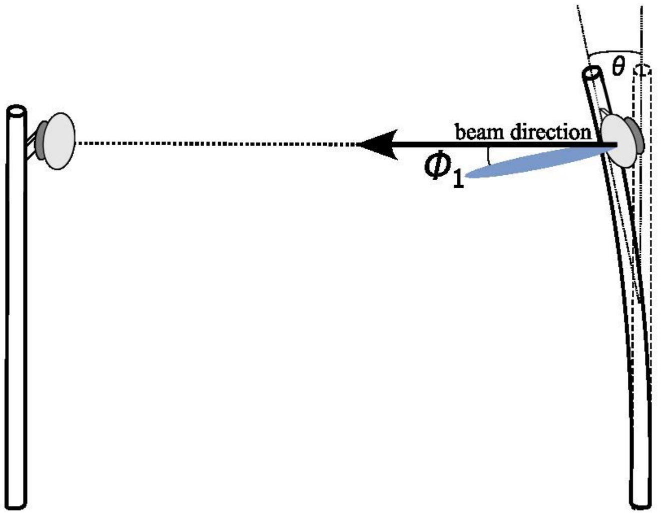

Figure S1 Beam misalignment angle due to pitch inclination

Thus the attenuation is $A_1 = g(\varphi_1) = g(\theta)$ where g is the antenna attenuation function. (Equation 12 of the manuscript)

However, for side inclinations, it can be shown by the diagram in Figure S2 that the beam misalignment angle (φ_2) is not equal to the pole inclination angle (θ), and is calculated by

$$\varphi_2 = \tan^{-1}\frac{l}{r_0}, \text{ where } l = L\ \sin\theta$$

where L is the pole length and r_0 is the path distance. Therefore the attenuation is

$$\therefore A_2 = 2 \cdot g(\tan^{-1}\frac{L\sin\theta}{r_0})$$

For $L \ll r_0$, the beam misalignment angle is much smaller in the side inclination case ($\varphi_2 \ll \theta$).

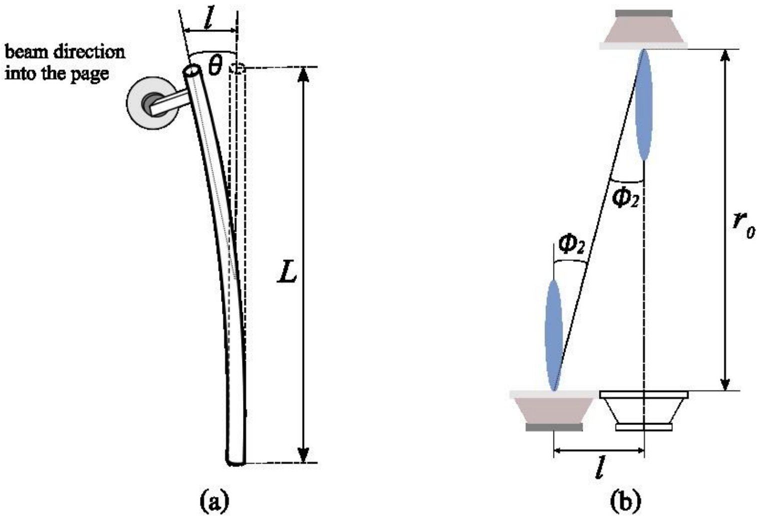

Figure S2 Beam misalignment angle due to side inclination (a) view from the back of antenna (b) view from the top

When we plot the attenuation as the function of the pole inclination angle in the two directions (pitch and side), for the path length r_0=150 m. The side inclination caused lower attenuation by 2 orders of magnitude, as in Figure S3 below.

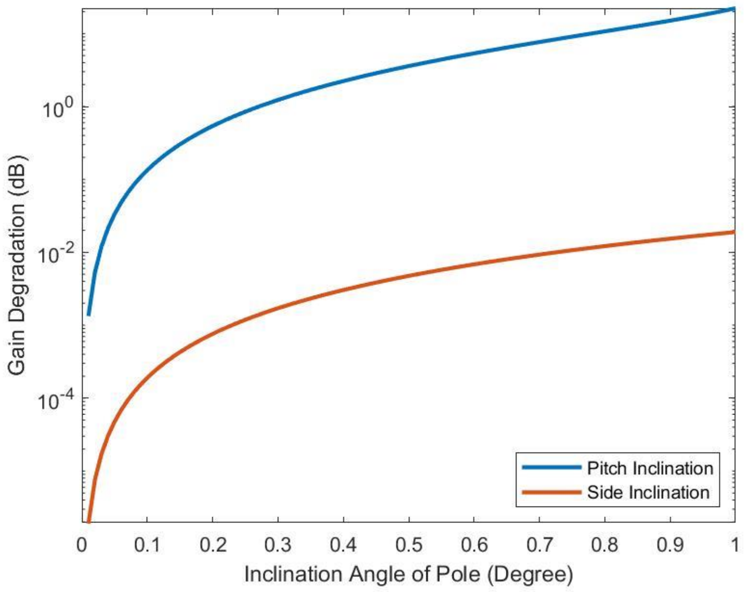

Figure S3 Comparison of gain degradation by pitch and side inclinations

2. Fresnel zone clearance analysis

The diagram of the link, including buildings between the path, is shown in Figure S4. The elevations of both antennas are approximately equal, and the earth curvature can be ignored at this short path distance (150m).

Multipath effect can be neglected in the experiment since the calculated primary Fresnel zone is completely clear of obstructions, in Table S1.

Table S1 Primary Fresnel zone clearance data.

Fresnel zone radius at 3 locations containing buildings	a_1 = 0.089 m	a_2 = 0.388 m	a_3 = 0.040 m
LOS clearance	b_1 = 0.700 m	b_2 = 7.0 m	b_3 = 0.600 m
Fresnel zone clearance	0.611 m	6.612 m	0.56 m

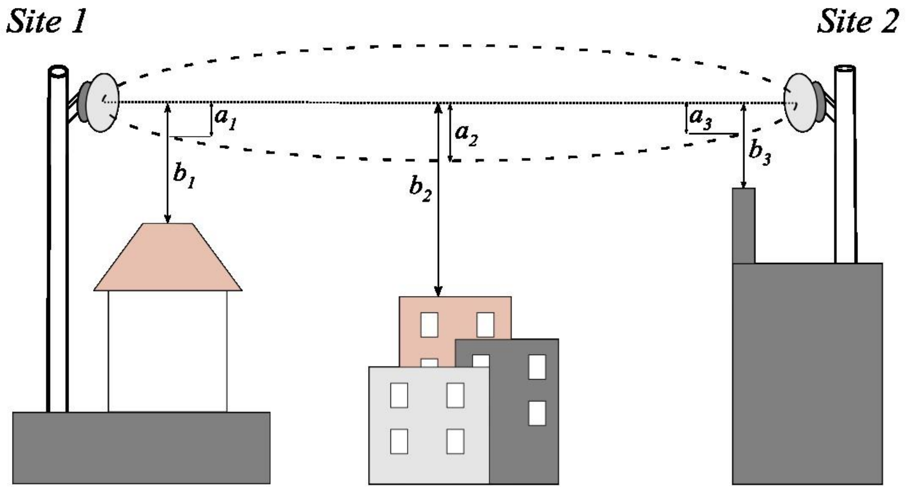

Figure S4 Diagram of the actual link and existing buildings between the path.

Note: The calculation of Fresnel zone radius. (The first Fresnel zone, n=1)

$$r^2 = n\frac{c}{f}\frac{d_1 \cdot d_2}{d_1 + d_2}$$

where d_1 is the horizontal distance between the potential obstruction and the first antenna, and d_2 is the horizontal distance between the potential obstruction and the second antenna. The total distance is r_0 = 150m.

Fresnel zone radius of the first building at d_1 = 2 m, d_2=150-d_1=148 m

$$r = \sqrt{\frac{3 \times 10^8}{74.625G} \times \frac{2 \cdot (150 - 2)}{2 + 148}}$$
$$r = 0.089\,m$$

Fresnel zone radius around the middle point of the path (the approximate location of the middle building) $d_1 = d_2 = \frac{r_0}{2}$

$$r = \sqrt{\frac{3 \times 10^8}{74.625G} \times \frac{(\frac{150}{2})(\frac{150}{2})}{150}}$$
$$r = 0.388\,m$$

Fresnel zone radius at site 2 (a_3) d_1=149.4m, d_2 = 150-d_1=0.6 m

$$r = \sqrt{\frac{3 \times 10^8}{74.625G} \times \frac{(150 - 0.6) \cdot 0.6}{150}}$$
$$r = 0.040\,m$$
